# Neutrophil in the suppressed immune microenvironment: Critical prognostic factor for lung adenocarcinoma patients with KEAP1 mutation

**DOI:** 10.3389/fgene.2024.1382421

**Published:** 2024-06-19

**Authors:** Zhongzhao Wang, Haojue Wang, Mingjia Liu, Xinhang Ning, Yang Chen, Hao Tang

**Affiliations:** ^1^ Department of Respiratory and Critical Care Medicine, Changzheng Hospital, Naval Medical University, Shanghai, China; ^2^ School of Basic Medicine, Second Military Medical University (Naval Medical University), Shanghai, China

**Keywords:** neutrophil, NSCLC, tumour microenvironment, lung adenocarcinoma, KEAP1

## Abstract

**Purpose:**

It is still unclear whether KEAP1 mutation is detrimental to immunotherapy of lung adenocarcinoma (LUAD) patients, we try to analyse the exact changes in the TME in LUAD patients with KEAP1 mutations and to identify key factors influencing prognosis.

**Experimental design:**

A total of 1,029 patients with lung squamous carcinoma (LUSC) or LUAD with data obtained from The Cancer Genome Atlas were included in this study. The TME and OS of patients with LUAD stratified by mutant *versus* wild-type KEAP1 status were comprehensively measured. Moreover, we classified LUAD patients with KEAP1 mutations into three subtypes, by unsupervised consensus clustering. We further analysed the TME, OS, commutated genes and metabolic pathways of different subgroups. A total of 40 LUAD patients underwent immunotherapy were collected and classified into mutant KEAP1 group and wild-type KEAP1 group. We also conducted immunohistochemical staining in KEAP1-MT groups.

**Result:**

Suppressed TME was observed not only in LUAD patients but also in LUSC patients. LUAD patients with mutant KEAP1 underwent immunotherapy had worse PFS than wild-type KEAP1. Unsupervised consensus clustering analysis suggested that the three subtypes of patients exhibited different densities of neutrophil infiltration and had different OS results: cluster 2 patients had significantly higher levels of neutrophils had significantly worse prognoses than those of patients in clusters 1 and 3 and patients with wild-type KEAP1. Univariate and multivariate Cox analyses proved that a high density of neutrophils was significantly associated with worse OS and immunohistochemical staining proved that shorter PFS showed high density of neutrophils.

**Conclusion:**

KEAP1 mutation significantly suppresses the tumour immune microenvironment in LUAD patients. LUAD patients with mutant KEAP1 underwent immunotherapy had worse PFS than with wild-type KEAP1. Neutrophils may play an important role in the prognosis of LUAD patients with KEAP1 mutations and may provide a promising therapeutic target.

## Introduction

Lung cancer is still the leading cause of mortality related to cancer worldwide (Mamdani et al., 2022). Non-small-cell lung cancer (NSCLC) is the most prevalent type of lung cancer, accounting for more than 85% of cases (Gong et al., 2020). NSCLC is mainly divided into lung adenocarcinoma (LUAD), lung squamous carcinoma (LUSC) and other rare subtypes (Wang, Herbst, and Boshoff, 2021). Despite remarkable improvements in lung cancer treatments, including targeted therapy and immunotherapy, less than 25% of NSCLC patients have been shown to benefit from targeted therapy in recent decades (Boumahdi and de Sauvage, 2020).

Recent studies have shown that the occurrence of mutations in the Kelch-like ECH-associated protein 1 (KEAP1) in NSCLCs is up to 20%. KEAP1 plays a critical role in the regulation and management of the oxidative stress pathway by negatively regulating nuclear factor erythroid-2-related Factor 2 (NRF2) (Baird and Yamamoto, 2020). In several studies, KEAP1 mutation has been found to be related to chemotherapy resistance, while understanding of the influence of KEAP1 mutation on immunotherapy in NSCLC patients is still controversial (Zhu et al., 2021).

In a retrospective cohort study of over 1,200 patients from multiple centres, KEAP1 and KRAS commutations were shown to confer worse results in LUAD patients, but KEAP1 mutations alone without KRAS commutation did not (Ricciuti et al., 2021). Nevertheless, in another prospective cohort study of up to 800 patients, patients with KEAP1 mutation had worse outcomes following immunotherapy (Zhu et al., 2021). Similarly, a retrospective cohort study by Mohamed et al. including more than 5,000 patients showed that KEAP1 or TP53 mutations led to a negative prognosis (Saleh et al., 2022).

In recent years, the tumour immune microenvironment (TME) has been considered a potential prognostic factor for the treatment of NSCLC patients, and some researchers have tried to alter the TME as an adjuvant therapy to reduce the resistance to ICS and even as an independent therapy to improve objective survival (OS) (Genova et al., 2022). Increasing evidence demonstrates that a high density of CD8^+^ and CD4^+^ T lymphocytes in tumours is an independent predictor for a worse prognosis (Brambilla et al., 2016). In previous studies, a low density of tumour-infiltrating lymphocytes was found to lead to worse efficacy of immunotherapy and resistance to ICS(Donnem et al., 2015). In contrast, in Bretscher’s hypothesis, type 2 T helper cells (Th2) and T regulatory cells (Tregs) were identified as determinants of NSCLC and were considered to be factors that promote cancer progression (Hamilton and Bretscher, 2008). Similarly, a small retrospective study of 11 samples showed that an increased Th1 to Th2 ratio was associated with a higher density of tumour-infiltrated CD8^+^ T cells (Gentles et al., 2016).

In recent years, understanding of the role of neutrophils in tumours has undergone an overwhelming transformation (Aloe et al., 2021). Emerging evidence suggests that neutrophils are major predictors of poor outcomes in NSCLC patients (Gentles et al., 2016). Previous studies have demonstrated that a high density of neutrophils in tumour tissue correlates with poorer prognosis than that observed for patients with a low neutrophil density (Zhang et al., 2021). Additionally, tissue-resident neutrophils derived gene signature were proved associated with failure of immunotherapy in NSCLC patients (Salcher et al., 2022). Nevertheless, the exact mechanism of how neutrophils influence the progression, metastasis and treatment of cancer remains unknown (Coffelt and Carlin, 2019). It is also unclear how tumours affect neutrophil trafficking.

To identify determinants of the prognosis of LUAD patients with KEAP1 mutations in the TME, we performed a comprehensive analysis of the TME and OS of LUAD and LUSC patients. Additionally, we classified LUAD patients with KEAP1 mutations into three subtypes, clusters 1, 2 and 3, by unsupervised consensus clustering. Furthermore, we analysed the TME, OS, commutated genes and metabolic pathways of cluster 2 and clusters 1 and 3 patients and LUAD patients with wild-type KEAP1.

## Methods and materials

### Collection of clinical samples

A total of 40 patients who were confirmed lung adenocarcinoma pathologically underwent immunotherapy in respiratory medicine department of Shanghai Changzheng hospital from January 2018 to December 2022 were collected, which was named CZHR cohorts. According to the results of NGS (Next-generation sequencing), Patients were classified into KEAP1-WT groups (n = 30) and KEAP1-MT (n = 10), (Table 2). All patients were diagnosed and evaluated Performance status score (PS) and TNM score according to eighth edition of American Joint Committee on Cancer (AJCC). Inclusion criteria were listed: 1) no other anti-cancer therapy received before immunotherapy. 2) patients were conducted NGS which included KEAP1 and PD1/PDL1 test. 3) patients were confirmed lung adenocarcinoma pathologically. The exclusion criteria were as listed: 1) patients lost to follow up. 2) patients were not received regular immunotherapy and imageological examination. This retrospective study was approved by the Ethical Committee of Changzheng Hospital, Navy Medical University. This study’s use of human subjects complies with the Declaration of Helsinki.

### Acquisition of raw data

Somatic mutation data were obtained from R-Pack TCGAbiolinks (Colaprico et al., 2016) from Bioconductor. The MAF format files of LUAD and LUSC tumour cohorts in TCGA were obtained by using the R package TCGAbiolinks, and the samples of KEAP1 gene mutation (n = 90 in LUADs, n = 50 in LUSCs) in the MAF file were extracted for follow-up research. Tumour samples of TCGA LUAD patients (n = 513) and TCGA LUSC patients (n = 498) were extracted from the RNAseq data in the UCSC Xena database (https://xenabrowser.net/datapages/) (Goldman et al., 2020)in the transcript per million (TPM) format of TCGA and GTEx data portal (The GTEx Consortium et al., 2015) uniformly processed by the toil process (Vivian et al., 2017), and the TPM was processed by log2. The clinical information corresponding to all samples came from TCGA. clinical characteristics of patients with LUAD from TCGA were listed in Table1.

**TABLE 1:** Characteristics of patients with adenocarcinoma of lung in TCGA.

Characteristics		KEAP1 wt (n = 437)	KEAP1 mt (n = 94)	Overall (531)
Age (%)		65.40	64.68	65.28
	≥60	307 (70.3%)	63 (67%)	370 (69.7%)
	<60	113 (25.8%)	29 (30.9%)	142 (26.7%)
	NA	17 (3.9%)	2 (2.1%)	19 (3.6%)
Sex (%)	Female	246 (56.3%)	37 (39.4%)	283 (53.3%)
	Male	191 (43.7%)	57 (60.6%)	248 (46.7%)
Smoking index		41.33	43.05	41.67
Stage (%)				
	NA	6 (1.4%)	2 (2.1%)	8 (1.5)
	Stage I	5 (1.1%)	0	5 (0.9%)
	Stage IA	110 (25.2%)	25 (26.6%)	135 (25.4%)
	Stage IB	122 (27.9%)	20 (21.3%)	142 (26.7%)
	Stage II	1 (0.2%)	0	1 (0.2%)
	Stage IIA	42 (9.6%)	9 (9.6%)	51 (9.6%)
	Stage IIB	63 (14.4%)	10 (10.6%)	73 (13.7%)
	Stage IIIA	63 (14.4%)	14 (14.9%)	77 (14.5%)
	Stage IIIB	8 (1.8%)	3 (3.2%)	11 (2.1%)
	Stage IV	17 (3.9%)	11 (11.7%)	28 (5.3%)

### Immune infiltration

First, the TPM data of immune regulation-related genes were extracted and tested for differences between mutant and wild-type KEAP1 patients. Furthermore, using the R package gene set variation analysis (GSVA) (Hänzelmann, Castelo, and Guinney, 2013), single-sample GSEA was performed using the TPM data of immune cell biomarkers (Shen, Peng, and Shen, 2020) to determine the infiltration of each immune cell in each sample, and the differences between mutant and wild-type KEAP1 patients were assessed. After obtaining consistent clustering results, the same method was used across different subtypes to measure and compare levels of immune cell infiltration among different subtypes.

### Evaluation of prognostic efficacy

We extracted the OS results and OS time corresponding to each sample. For patients with lung cancer, including those with LUADs and LUSCs, we used the R packages survival and survminer to perform the log rank test function to analyse patient survival and draw the survival curve. After obtaining consistent clustering results, we further analysed the survival of patients in different clusters using the same approach for both LUAD and LUSC patients to determine the impact of each cluster on the prognosis of lung cancer. To further evaluate the impact of the consistent clustering results on prognostic efficacy, we included the clustering results for type 2 T helper cell and neutrophil marker genes together with OS status and OS time into Cox regression for analysis and drew a forest map.

### Identification of differentially expressed genes

Based on the mutation results provided in MAF files, samples of wild-type KEAP1 and mutant KEAP1 were extracted from LUAD and LUSC patients, respectively, and grouped to find the genes differentially expressed between mutant KEAP1 and wild-type KEAP1 patients. The R package limma (Ritchie et al., 2015)was used to construct the comparison matrix for the two cohorts of patients with two types of lung cancer, fit the linear model, and extract the difference analysis results. In this analysis, we set the threshold of differentially expressed genes as fold change >2 and adjusted *p* < 0.05. The differentially expressed genes identified were used for subsequent consistent clustering. Differential analysis was also carried out through the above methods among the different patient subtypes obtained through consistent clustering, and the intersection of the differentially expressed genes between different subtypes and the differentially expressed genes of patients with KEAP1 mutation was taken. Considering that there were few intersecting genes, we appropriately reduced the threshold for identifying differential genes within a reasonable range (fold change >1.5 and adjusted *p* < 0.05).

### Enrichment analysis of differentially expressed genes

After the ID conversion of differentially expressed genes (DEGs) by the R package org. Hs.e.g.,.db, the upregulated genes were used for GO and KEGG enrichment analysis by using the R package clusterProfiler (Yu et al., 2012) to obtain relevant GO terms and KEGG pathways. All GO and KEGG results were filtered, and the results that met the conditions adjusted *p*-value <0.05 and Q value <0.2 were finally retained. The results were visualized using the R package ggplot2.

### Unsupervised consensus clustering

The R package ConsensusClusterPlus (Wilkerson and Hayes, 2010) was used to cluster the upregulated genes of LUADs and LUSCs in KEAP1 mutation samples. In this study, the specific parameters were (maxK = 10, reps = 1,000, pItem = 0.95, pFeature = 1, clusterAlg = “PAM”, corUse = “complete. OBS”, seed = 145252945).

### Correlation analysis

We extracted the marker genes of neutrophils and type 2 T helper cells corresponding to cluster 2 samples, the genes related to the nitrogen metabolism pathway, and the TPM of the intersection of upregulated genes between KEAP1 mutation and cluster 2 patients and conducted Spearman correlation analysis to assess whether there was an obvious correlation between the above three types of genes to determine whether there might be a mutual regulatory relationship among them. The correlation results were plotted using the R package CoxplexHeatmap (Gu, Eils, and Schlesner, 2016) and circlize (Gu et al., 2014) to draw a correlation heatmap.

### Immunohistochemical staining

Immunohistochemical staining of issue from lung cancer reveals different density of neutrophile in KEAP1-MT group (n = 10) with different PFS. Slides of tissue were hydrated and deparaffinized in xylene in serial alcohol solutions. The slides were then incubated with Ly6G (Lymphocyte antigen 6 complex locus G6D) antibody for neutrophils. All slides were counterstained with hematoxylin (Roche), cover-slipped, and finally digitized using a multispectral imaging platform (PhenoImager HT, Akoya). Neutrophil counts were calculated randomly intercepting five squares with an area of 1 ${mm}^{2}$ from each immunohistochemical stain slice and averaging the number by two pathologists. According to the manufacturer’s instructions, PD-L1 tumour proportion score (TPS) Immunohistochemistry was performed using the PD-L1 kit (PD-L1 IHC 22C3 pharmDX; Dako, Carpinteria, CA, United States).

### Statistical analyses

Using R version 4.1.0, after the Shapiro–Wilk normality test, the TPM data of immune response-related genes and marker gene sets of immune cells in mutant and wild-type KEAP1 patients with LUAD and LUSC samples did not meet normality (*p*-value >0.05), so a nonparametric test (Shapiro–Wilk normality test) was used to assess differences. A *p*-value <0.05 was considered a statistically significant difference. The chi square test was used to determine the difference in the frequency of mutant genes between cluster 2 and non-cluster 2 samples.

## Results

### Differences in the immune microenvironment of NSCLC between patients with KEAP1 mutation and wild-type KEAP1

**FIGURE 1:**
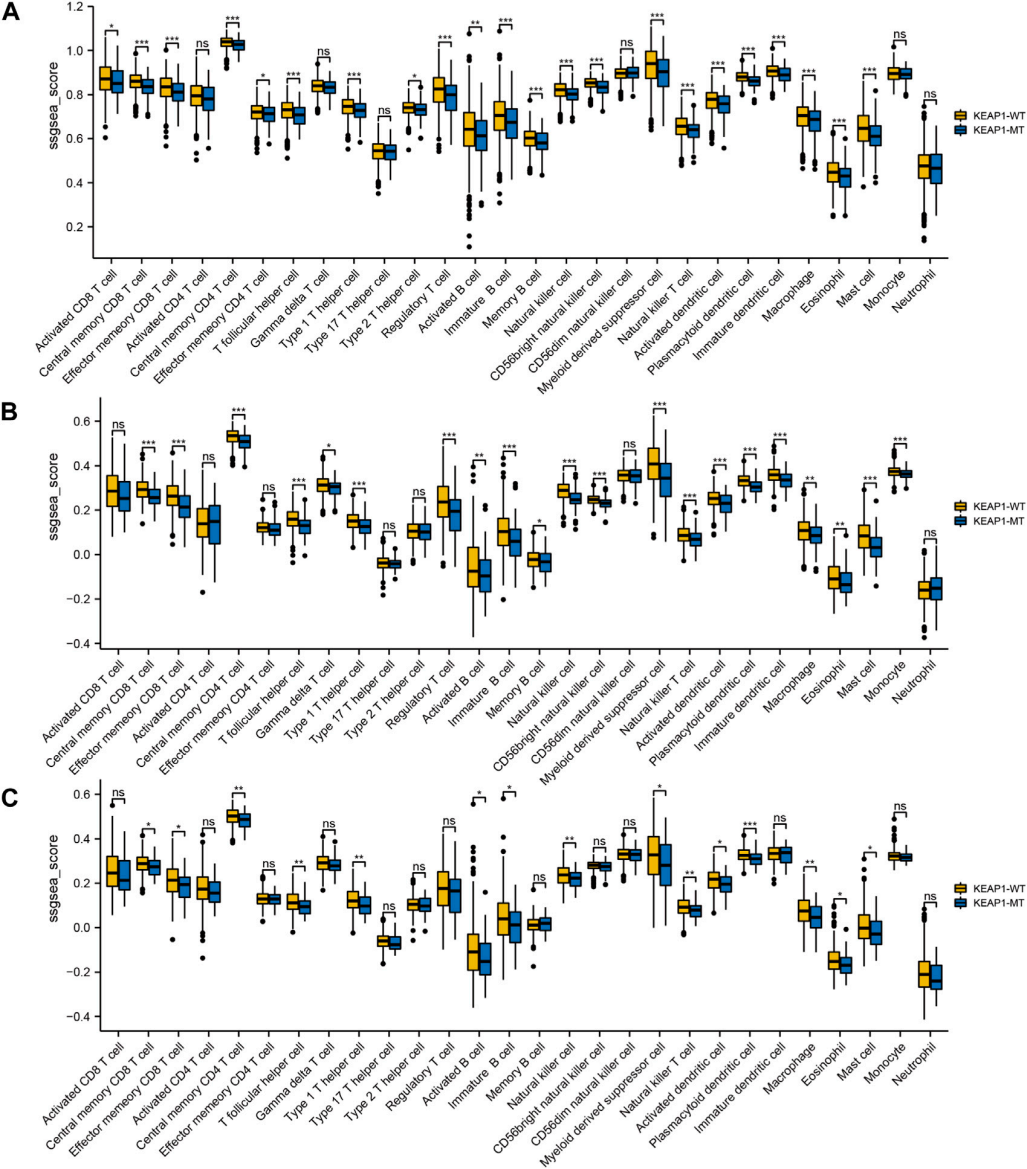
The level of ssGSEA of immune cells in lung cancer samples in TCGA is tested by Wilcox between Keap1 mutation group (KEAP1-MT) and wild group (KEAP1-WT). **(A)**. Comparison of immune infiltration levels between Keap1 mutant group and wild group in combined LUAD and LUSC samples. **(B)**. Comparison of immune infiltration levels between KEAP1-MT and KEAP1-WT in LUAD samples. **(C)**. Comparison of immune infiltration levels between KEAP1-MT and KEAP1-WT in LUSC samples. (“ns” means *p* ≥ 0.05; “*“means *p* < 0.05; “**“means *p* < 0.01; “***” means *p* < 0.001).

We firstly performed a comprehensive analysis of the TME of all LUAD and LUSC patients’ samples. Overall, the infiltration level of almost all immune cells in the tumour samples with KEAP1 mutation was lower than that of the wild-type samples, and most of them were statistically significant. We further analyzed the infiltration level of immune cells in LUADs and LUSCs, respectively. In LUADs, the immune cell infiltration level of KEAP1 mutant samples decreased more significantly, especially in central memory CD8 T cell, Effector memory CD8 T cell, Central memory CD4 T cell, T follicular helper cell, Type 1 T helper cell, Regulatory T cell, Natural killer cell, CD56bright natural killer cell, Myeloid derived suppressor cell, Natural killer T cell, Activated dendritic cell, Plasmacytoid dendritic cell, Immature dendritic cell, Mast cell and Monocyte, whose *p* values were less than 0.001. While, significant differences of neutrophil were observed neither in LUADs nor LUSCs (Figure 1).

### Effect of KEAP1 mutation on the prognosis of patients with lung cancer

**FIGURE 2:**
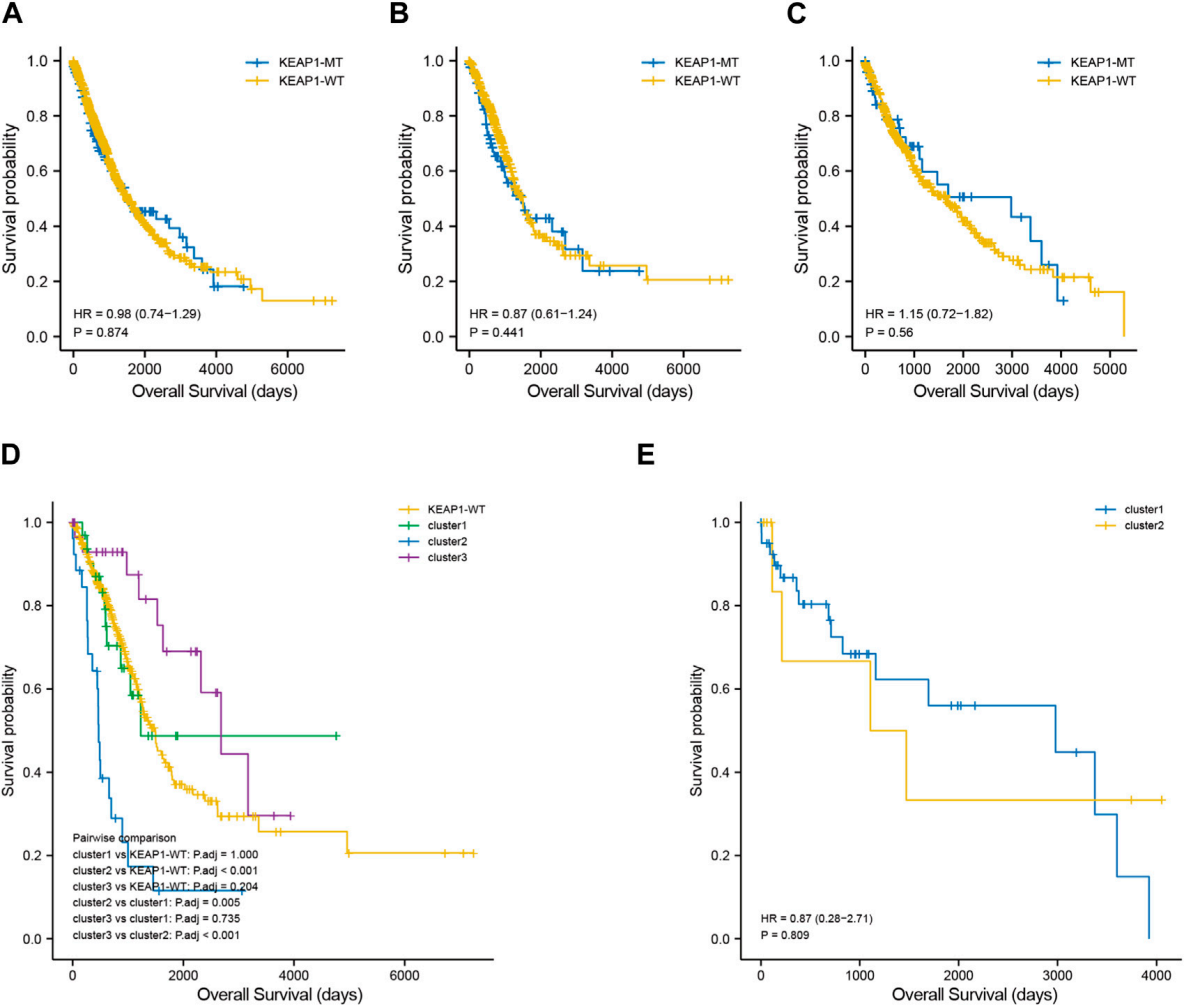
Kaplan Meier curve shows the difference of overall survival rate among different groups. **(A–C)**. survival curves between KEAP1 mutant samples and KEAP1 wild-type samples in lung cancer samples, LUAD samples and LUSC samples. **(D)**. Survival curves of three types of clusters and KEAP1 wild-type samples in LUADs. **(E)**. Survival curves of two types of clusters in LUSCs.

To explore the significance of KEAP1 mutation on the prognosis of lung cancer patients, we analysed the survival of lung cancer patients with wild-type KEAP1 and KEAP1 mutations. First, the overall analysis of all lung cancer samples obtained from TCGA revealed that the survival curve could not be significantly separated, and the *p*-value was not statistically significant (*p* = 0.874) (Figure 2A). We further divided lung cancer samples into LUADs and LUSCs for survival analysis. However, the results of Kaplan-Meier analysis of survival were also unsatisfactory (*p* values were 0.441 and 0.560, respectively) (Figure 2B,C). Generally, KEAP1 mutation did not show high prognostic value in the two types of lung cancer patients.

### Screening the subtypes with poor prognosis related to KEAP1 mutation by consistent clustering

Although the preliminary analysis did not indicate the prognostic value of KEAP1 mutation, according to previous research and experience, KEAP1 mutation has been shown to have an impact on the prognosis of lung cancer patients. Therefore, we further analysed the prognosis of patients with KEAP1-mutated samples through consistent clustering. To make the classification result based on the influence of KEAP1 mutation, we only included the differential genes identified with KEAP1 mutation (fold change >2, adjustment *p* < 0.05). After selecting for KEAP1 mutation, 98 and 57 upregulated genes in LUADs and LUSCs, respectively, were included in consistent clustering. According to the results obtained using the R package ConsensusClusterPlus, we selected K values that were evenly distributed and clearly distinguished in the consumption matrix. In this analysis, k = 3 LUADs and K = 2 LUSCs. Based on KEAP1 mutation, LUADs and LUSCs can be divided into 3 and 2 subtypes (Figure 2D,E), respectively. However, in LUSCs, there was no significant difference in prognosis between the two subtypes (*p* = 0.81) (Figure 2E). Fortunately, there was a reasonable prognostic difference between the three subtypes of LUAD patients (*p* < 0.001) (Figure 2D). Among them, the Kaplan–Meier curve of cluster 2 patients showed poorer prognoses than those of patients in clusters 1 and 3. We further included the immune cell infiltration level and OS of patients with KEAP1 mutation samples in Cox univariate and multivariate regression analyses. In univariate Cox regression analysis, subtype cluster 2 (*p* < 0.001) and the immune infiltration levels of active B cells (*p* = 0.035) and neutrophils (*p* = 0.006) were statistically correlated with prognosis. In the results of multivariate Cox regression analysis, cluster 2 (*p* = 0.013) and the infiltration levels of active B cells (*p* = 0.004) and neutrophils (*p* = 0.04) were independent risk factors after KEAP1 mutation (Figure 4A,B).

### Type of mutation in KEAP1 and Enrichment of related differentiated genes after KEAP1 mutation in LUADs

**FIGURE 3:**
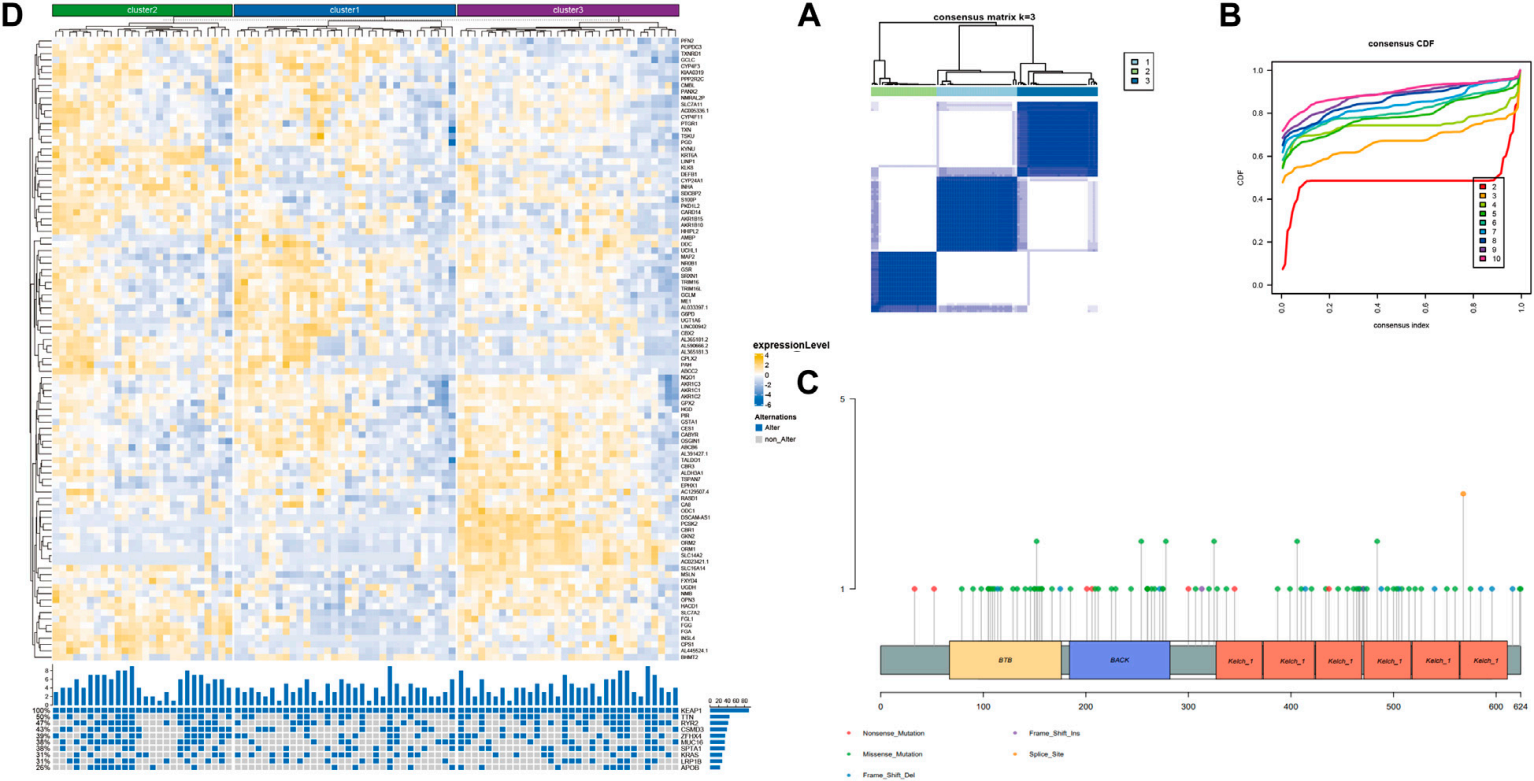
Unsupervised consistency clustering is carried out through concensusclusterplus. **(A)**. Consistency clustering matrix of LUAD mutation samples. **(B)**. Consistency clustering CDF diagram of LUAD mutation samples. **(C)**. Lollipop chart showed different KEAP1 mutation sites in LUADs. **(D)**. The subtype expression results obtained by LUAD consistent clustering and the gene mutation of the top 10 mutation variables in cluster2.

Mutation of KEAP1 occurred throughout the whole length of the protein (Figure 3C, supplementary table 2). Most of KEAP1 mutation in LUADs were missense mutation, which were also proved in the following GSEA (gene set enrichment analysis) (supplementary figure 1 C). In GSEA, ERBB2, MEK and NFE2L2 were up in KEAP1 mt group of LUADs. Enrichment of MF (molecular function), BP (biological process) and CC (cell component) were conducted according to gene ontology (GO) and Gene pathways were conducted according to KEGG (Kyoto Encyclopedia of Genes and Genomes). Antioxidant and activity and detoxification relevant function were significantly influenced in LUADs with KEAP1 mutation (supplementary figure 1A). Additionally, tumor-associated gene pathways including glutathione metabolism, chemical carcinogenesis, metabolism of xenobiotics by cytochrome P450 and cell adhesion molecules were significantly associated with KEAP1 mutation in LUADs (supplementary figure 1B).

### Differences in immune cell infiltration in different subtypes of LUAD identified with consistent clustering

**FIGURE 4:**
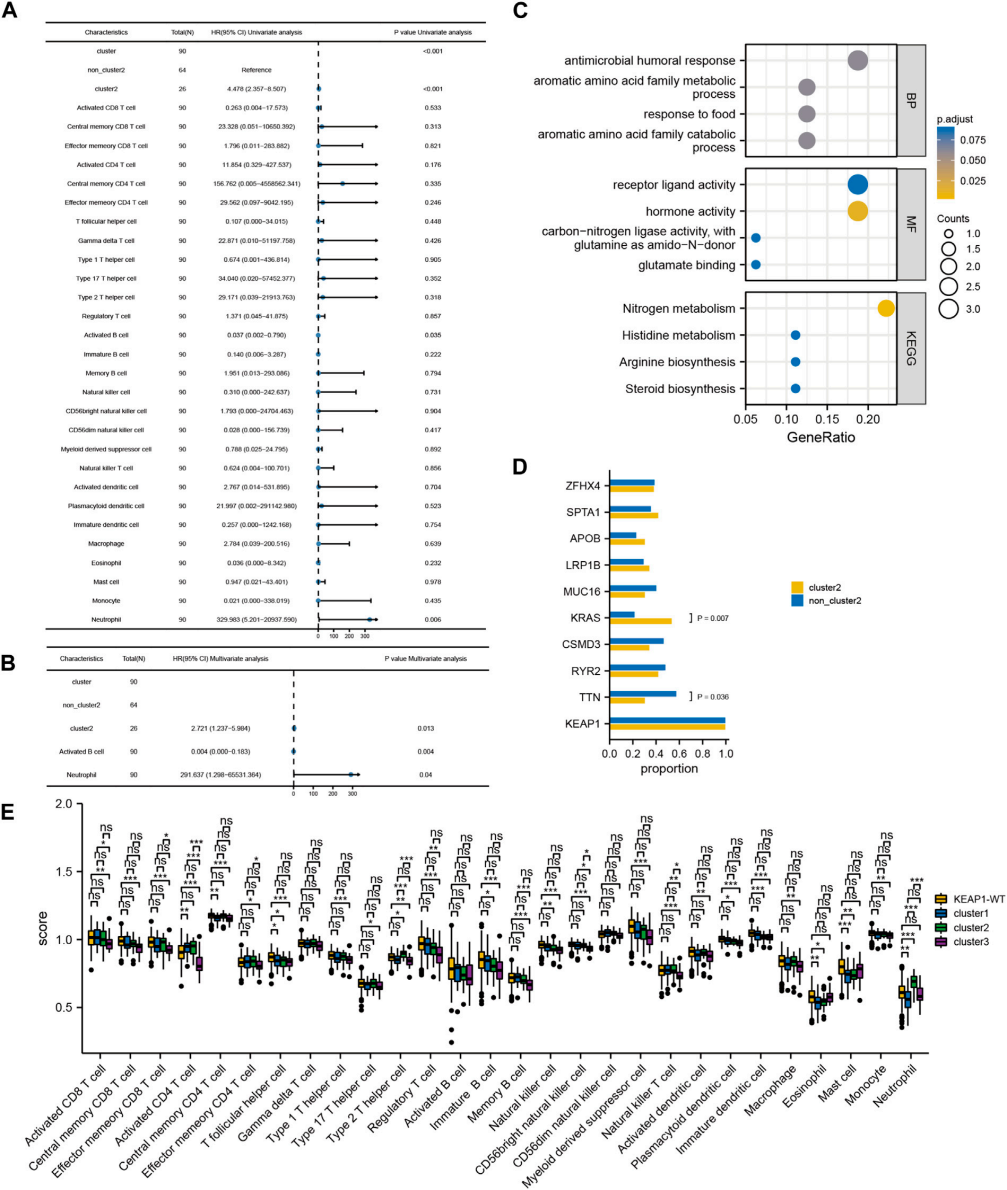
Cox regression analysis of immune infiltration level and consistency clustering on OS and the mechanism of poor prognosis of cluster2. **(A)**. Univariate Cox regression of the immune infiltration level and subgroups of consistency clustering. **(B)**. Multivariate Cox regression of the immune infiltration level and subgroups of consistency clustering. **(C)**. The enrichment and analysis of related differential genes between cluster2 and non-cluster2 group. **(D)**. Chi-square test results of TOP10 mutant genes among different subtypes. **(E)**. Comparison of immune cell infiltration levels between KEAP1 mutant samples and wild samples. (“ns” means *p* ≥ 0.05; “*“means *p* < 0.05; “**“means *p* < 0.01; “***” means *p* < 0.001).

**FIGURE 5:**
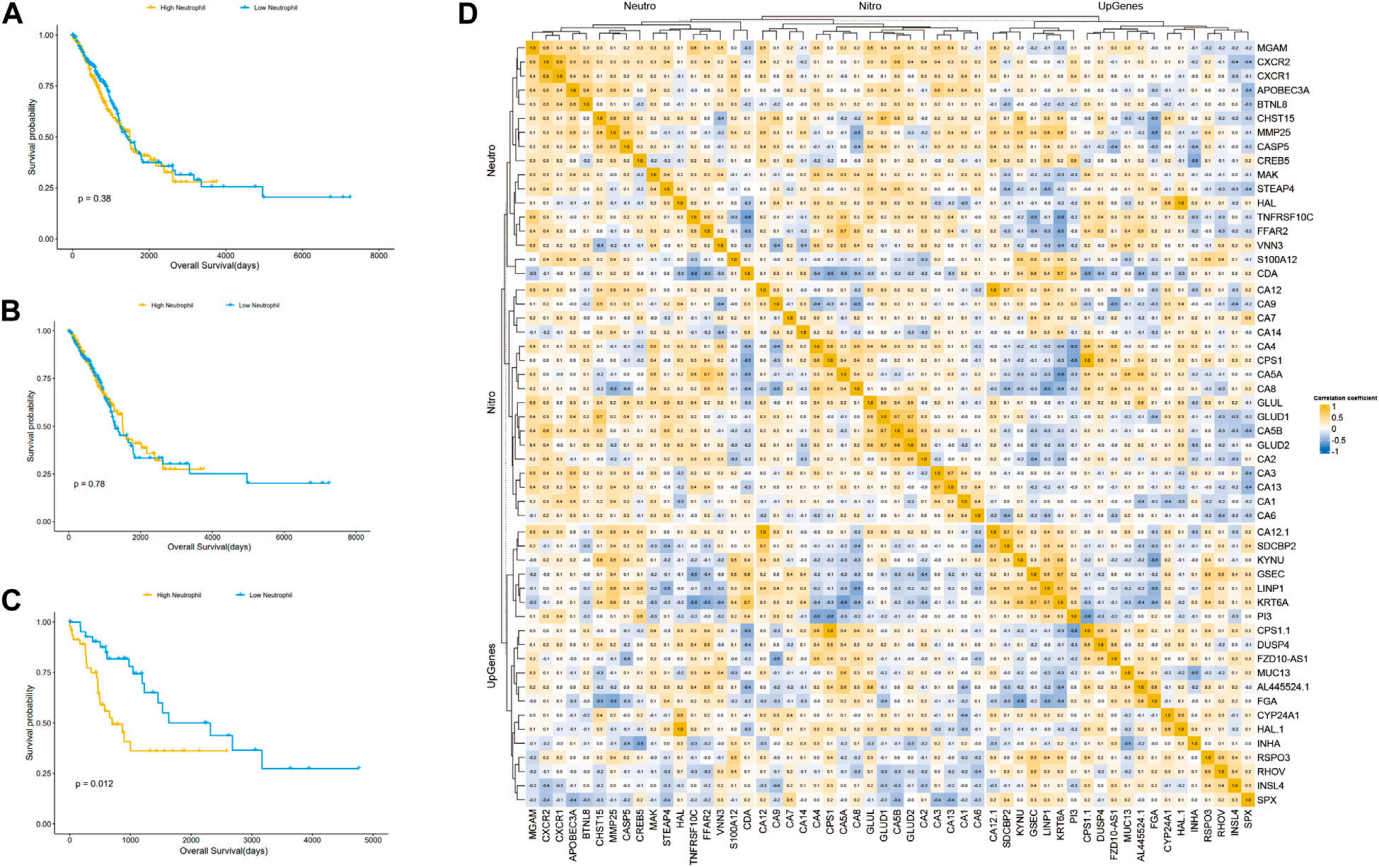
Kaplan Meier curve shows the difference of overall survival rate between high and low neutrophil in tumor immune microenvironment in different groups. **(A)**. The difference of OS between high and low neutrophil in tumor immune microenvironment in LUADs. **(B)**. The difference of OS between high and low neutrophil in tumor immune microenvironment in KEAP1 wt. **(C)**. The difference of OS between high and low neutrophil in tumor immune microenvironment in KEAP1 mt. **(D)**. Correlation heat map of KEAP1 mutation differential genes and cluster2 differential genes intersection genes, type 2 T helper cell, neutrophil marker genes and genes related to nitrogen metabolism pathway.

We speculated that the prognostic differences of patients in different LUAD subtypes might be mediated by different degrees of infiltration of specific immune cells. We used ssGSEA (single sample GSEA) to enrich and score the marker gene sets of different immune cells to determine the infiltration level of different immune cells. In general, the immune components of the three subtypes showed little difference, but it could be identified that in cluster 2, neutrophils and type 2 T helper cells had significant differences from those in cluster 1 and cluster 3 (*p* < 0.001), and the infiltration level was significantly higher in this group than in the other two subtypes (Figure 4E). The difference of overall survival rate between high and low neutrophil by set cutoff value as median in tumor immune microenvironment in different groups including LUADs, KEAP1 wt and KEAP1 mt groups were analysed. There is no significant difference in LUADs and KEAP1 wt groups (Figure 5A,B). However, in KEAP1 mt group, high neutrophil group showed significant better prognosis (*p* = 0.012) (Figure 4C).

### Mechanism of increased neutrophil infiltration in subtypes with poor prognosis

Previous studies have reported that neutrophils can help tumour cells better avoid the attack of T cells, resulting in a worse prognosis of lung cancer patients (Ponzetta et al., 2019). However, the mechanism of the increase in neutrophils in cluster 2 patients with KEAP1 mutation has not been explained. We aimed to assess the intersection between the differential genes of cluster 2 and cluster 1 and 3 patients and the differential gene of patients with KEAP1 mutation (fold change >1.5 and adjustment *p* < 0.05, the number of intersecting genes was 20). Through the enrichment analysis performed using the R package cluster profiler, only a few meaningful KEGG results and GO terms were obtained, including the nitrogen metabolism pathway (adjusted *p* = 0.0001) and the GO term hormone activity (adjusted *p* = 0.0002) (Figure 4C). Based on the enrichment results, we speculated that neutrophil infiltration in cluster 2 might be related to nitrogen metabolism. Therefore, we analysed the correlation between the expression of neutrophil marker genes, nitrogen metabolism pathway-related genes, cluster 2 upregulated genes and KEAP1 mutation intersection genes in cluster 2 samples (Figure 5D). We found that most of the genes related to the nitrogen metabolism pathway were highly correlated with neutrophil marker genes.

### Commutation of KEAP1 with TTN or KRAS leads to different prognoses of different patient subtypes

In addition, we also analysed the differences in the 10 most mutated genes in cluster 2 by the chi-square test. There were significant differences between the mutation rates of KRAS and TTN in cluster 2 and those in the other two subtypes (*p* values were 0.036 and 0.007, respectively). In the cluster 2 and noncluster 2 samples, the mutation rates of KRAS were 21.88% and 53.85% (Figure 4D), respectively, and the mutation rates of TNN were 30.77% and 57.81%, respectively. Therefore, we speculated that co-occurring KEAP1 and KRAS mutations might also lead to a worse prognosis of cluster 2 patients, while co-occurring KEAP1 and TNN mutations leads to a better prognosis of noncluster 2 patients.

### Effect of KEAP1 mutation on the lung adenocarcinoma patients in immunotherapy

**FIGURE 6:**
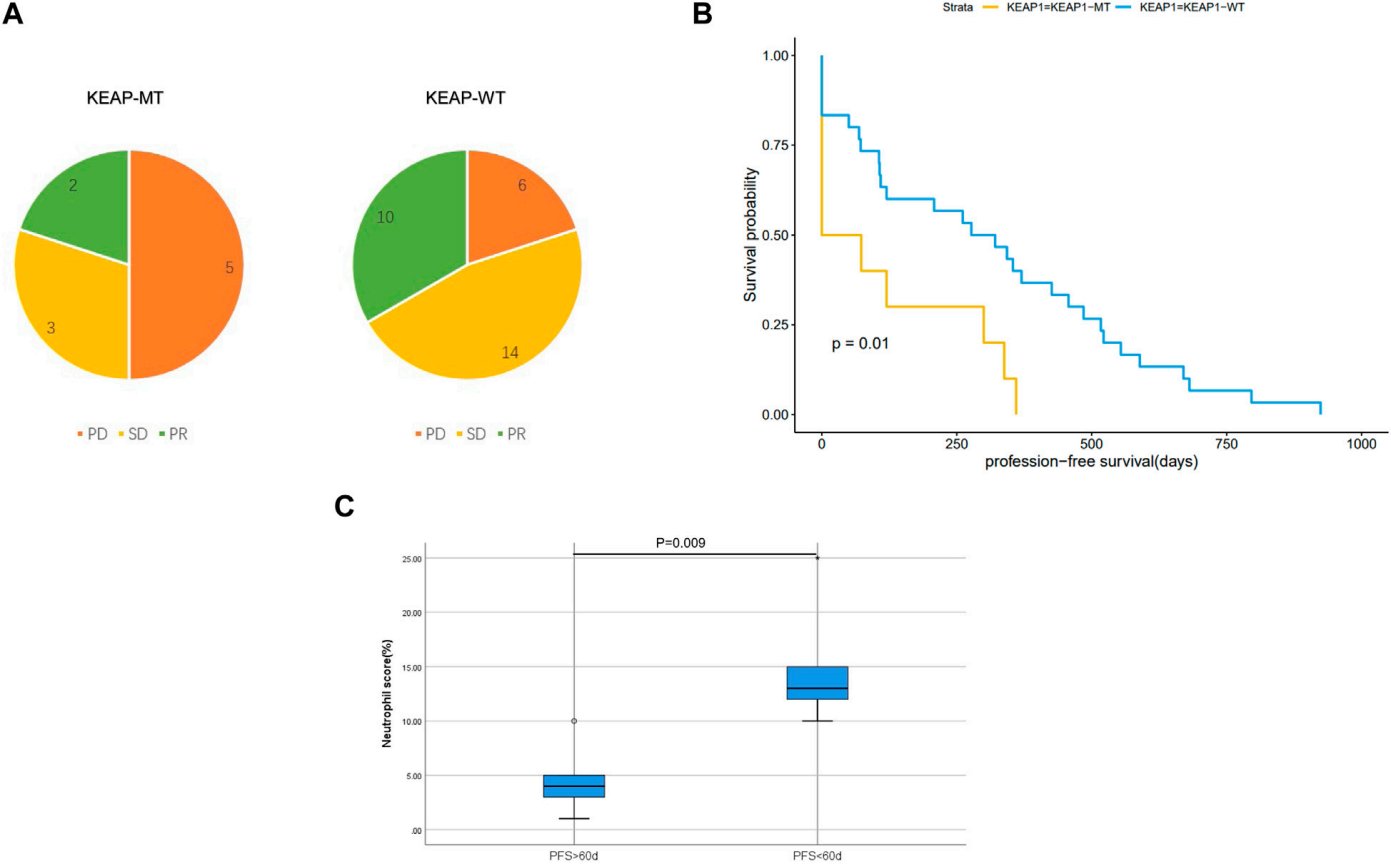
**(A)**. Sector diagram reveals difference of Best of response (BOR) among KEAP1-MT group and KEAP1-WT group of CZHR cohorts. **(B)**. Kaplan Meier curve shows the difference of progression-free survival (PFS) among KEAP1-MT group and KEAP1-WT group of CZHR cohorts. **(C)**. Immunohistochemical staining of tissue from lung cancer reveals different density of neutrophile granulocyte in KEAP1-MT group with different PFS.

We analysed characteristics of patients in CZHR cohorts and found no significant difference in PDL1/PD1 score, PS score, Stage and sex between KEAP1-MT group and KEAP1-WT group (Table 2). We further analysed Kaplan Meier curve among KEAP1-MT group and KEAP1-WT group of CZHR cohorts the difference of progression-free survival (PFS) and found KEAP1-MT group had significant worse prognosis than KEAP1-WT group of CZHR cohorts (*p* = 0.014) (Figure 6A). Similarly, Sector diagram revealed KEAP1-MT group had worse best of response (BOR) of CZHR cohorts. Nevertheless, *p*-value was 0.102, which might be caused by small sample size (Figure 6B). Furthermore, we conducted Immunohistochemical staining of tissue from lung cancer and metastatic lymph node carcinoma and revealed patients with PFS <60 days showed higher density of neutrophile than PFS> 60 days (4.6% VS 15% *p* = 0.009) (Figure 6C). Notably, we found that KEAP1 mutations seemed to occur in younger patient. (average age was 59.2 vs. 67.5; *p* = 0.005) and might be correlated with adrenal gland (*p* = 0.002) (Table 2).

**TABLE 2:** Characteristics of patients with adenocarcinoma of lung in CZHR cohorts.

Characteristics		KEAP1-MT (n = 10)	KEAP1-WT (n = 30)	*p*-value
Age (%)		59.2	67.5	**0.005**
	≥60	5 (50%)	27 (90%)	**0.015**
	<60	5 (50%)	3 (10%)	**0.015**
Sex (%)				0.307
	Female	0	6 (20%)	
	Male	10	24 (80%)	
Smoking history		8 (80%)	18 (60%)	0.446
Smoking index		944	1,021	0.585
Stage (%)				0.178
	I	0	0	
	II	0	6 (20%)	
	III	3 (30%)	9 (30%)	
	IV	7 (70%)	15 (50%)	
PS score				0.061
	0	7 (70%)	11 (36.7%)	
	1	3 (30%)	17 (56.6%)	
	2	0	2 (6.7%)	
	3	0	0	
	4	0	0	
metastasis		8 (80%)	15 (50%)	0.145
	pleura	3 (30%)	7 (23%)	0.689
	bone	5 (50%)	7 (23%)	0.133
	brain	0	2 (6.7%)	1
	adrenal gland	4 (40%)	0	**0.002**
	Other	1 (10%)	3 (10%)	1
PDL1 (TPS)	0	3 (30%)	8 (26.7%)	1
	>0	7 (70%)	22 (73.3%)	1
Best of response (BOR)				
	PD	5 (50%)	6 (20%)	0.103
	SD	3 (30%)	14 (46.7%)	0.471
	PR	1 (10%)	10 (33.3%)	0.693

Bold values were *p* < 0.05

### Lung adenocarcinoma KEAP1 mutated patients with different density of neutrophile granulocyte showed different responds in immunotherapy

**FIGURE 7:**
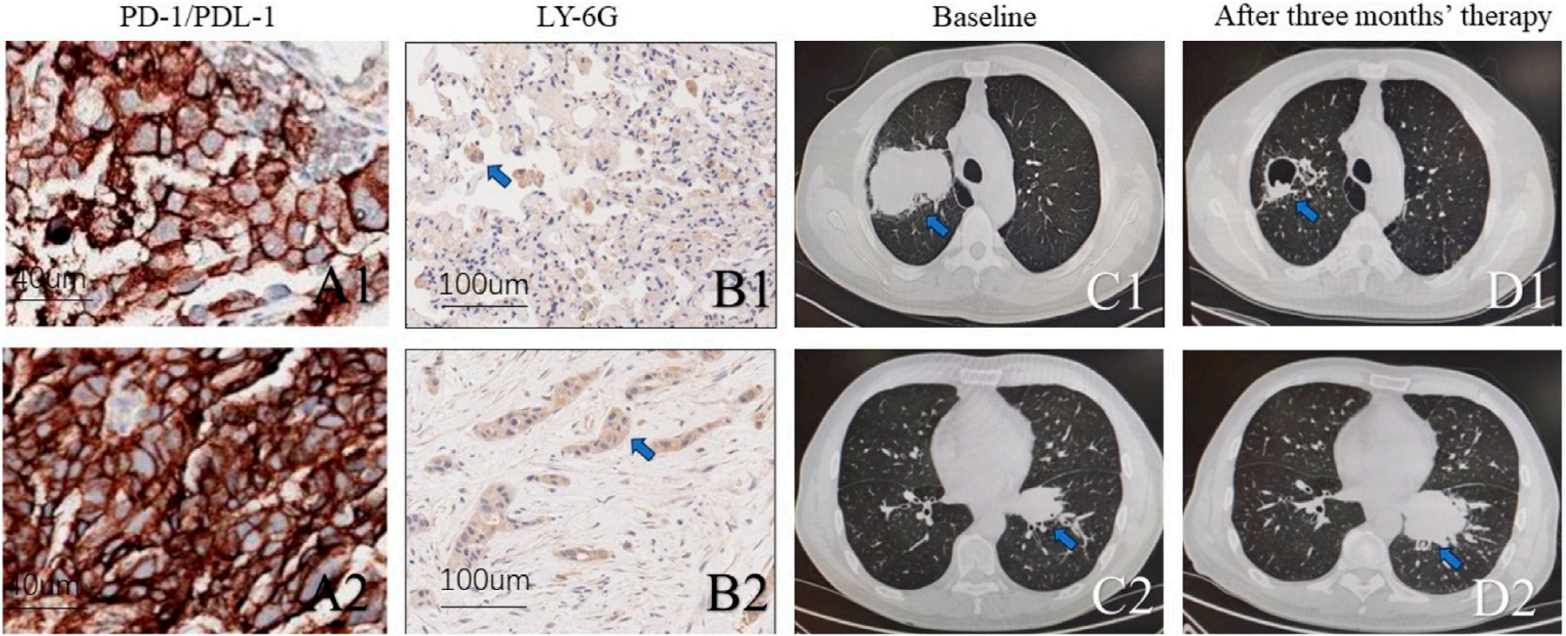
Two KEAP1 mutated patients with different density of neutrophile granulocyte received standard therapy of albumin paclitaxel + carboplatin + Coreda for 3 months and showed totally different responds. **(A1,A2)**. Photograph of PD-L1 and tumour proportion score (TPS) (B1,B2). Immunohistochemical staining of tissue from lung cancer reveals density of neutrophile granulocyte. **(C1, C2)**. Chest CT of pulmonary tumor at initial diagnosis. **(D1,D2)**. Chest CT of pulmonary tumor after receiving standard therapy of albumin paclitaxel + carboplatin + Coreda for 3 months.

Two lung adenocarcinoma patients with KEAP1 received standard therapy of albumin paclitaxel + carboplatin + Coreda for 3 months after tumour proportion score (TPS) of PD-L1 Immunohistochemical test showed 95% and 90% respectively (Figure 7A1,A2). Chest CT examination after 3 months’ treatment indicated that patients with high neutrophil density (Figure 7B1,B2) in tumor tissue had worse responds (Figure 7C1,D1,C2,D2). Conversely, The lung adenocarcinoma patient with KEAP1 mutation and lower density of neutrophile granulocyte significantly shrink in 3 months (Figure 7C1,D1).

## Discussion

In this study, we analysed the tumour immune microenvironment in LUAD and LUSC patients stratified by mutant and wild-type KEAP1 status using data obtained from the TCGA cohort. As a previous study showed (W. Cheng et al., 2021), we also found that mutant KEAP1 in LUADs could suppress the immune microenvironment. Moreover, similar changes were observed in LUSCs with KEAP1 mutations. However, mutant-type KEAP1 had a more obvious suppression of the tumour immune microenvironment in LUADs than in LUSCs. Levels of most immune cells, including a series of CD4^+^ and CD8^+^ T lymphocytes, B lymphocytes, macrophages, natural killer cells, eosinophils, dendritic cells, and monocytes, were lower in mutant KEAP1 LUADs. However, only approximately half of the immune cells exhibited statistically significant differences, including central and effector memory CD8^+^ cells, central memory CD4^+^ T cells, T follicular helper cells, type 1 helper cells, activated and immature B cells, natural killer cell myeloid-derived suppressor cell natural killer T cells, activated and plasmacytoid dendritic cells, macrophages, eosinophils, and mast cells, in LUSCs. Nevertheless, no statistical significance was found in levels of neutrophils in LUADs or LUSCs between mutant KEAP1 and wild-type KEAP1 samples.

In previous studies, it has been shown that a suppressed immune microenvironment is closely related to worse prognosis in NSCLC (Shi et al., 2022). Paradoxically, we analysed the OS of patients with and without KEAP1 mutations and found no statistically significant difference between them. Neither LUADs nor LUSCs were found to have a significant difference in OS between mutant KEAP1 and wild-type KEAP1 samples. Nevertheless, we found that KEAP1 patients had significant worse progression-free survival (PFS) than KEAP1-WT group in CZHR cohorts (N = 40) (*p*-value was 0.014). We speculated that different therapy in TCGA may contributed to the different results and different races also might put into consideration.

Nclear factor (erythroid-derived 2)-like 2 (NRF2) and its negative regulator, KEAP1 are frequently mutated in cancer, these mutations drive constitutive NRF2 activation and correlate with poor prognosis (Kerins and Ooi, 2018). NRF2 is a key regulator for cellular defense of intrinsic and extrinsic oxidative and electrophilic stimuli (Adinolfi et al., 2023). In our study, GSEA (gene set enrichment analysis) showed that NFE2L2 the gene encoding for NRF2 expression increased after KEAP1 mutation. In cancer, loss of function in KEAP1 results in NRF2 hyperactivity leading to a low density of lymphocytes and macrophages in TME (Härkönen et al., 2023). However, few studies on the interaction between KEAP1 mutation and neutrophils in the immune microenvironment were reported.

Mutations in KEAP1 occur throughout the length of the protein randomly (Kerins and Ooi, 2018), we also found that LUADs with KEAP1 mutation have different mutation sites (Figure 3D, Supplementary table2). However, there are few studies focus on function of KEAP1 mutation of different sites. We further explore enrichment and analysis of related differential genes between KEAP1 wt and KEAP1 mt. Our analysis suggested that immunomodulatory metabolites and enhanced antioxidant capacity might contribute to KEAP1-associated immune evasion. Enhanced antioxidant capacity and immunomodulatory metabolites in NRF2 Pathway was also emphasized in other studies (Fahrmann et al., 2022; Renken et al., 2022).

For the 90 LUAD patients with KEAP1 mutations in TCGA, unsupervised consensus clustering was conducted to divide all patients into three subgroups according to RNA expression. Intriguingly, in contrast with those of cluster 1, cluster 3 and LUADs with wild-type KEAP1 mutations, the OS of cluster 2 was significantly shorter (*p* < 0.001). To determine the characteristics and causes of worse prognosis in cluster 2 patients, we comprehensively analysed the tumour immune microenvironments of samples from the patient subgroups. The abundance of neutrophils and type 2 T helper cells in the tumour immune microenvironment of cluster 2 patients was obviously higher than those of patients in clusters 1 and 3. Increasing evidence has shown that the density of tumour-associated neutrophils (TANs) increases in NSCLC with tumour progression (Zhang et al., 2021).

Enrichment analysis of differentially expressed genes indicated that the nitrogen metabolism pathway might play an important role in the worse OS of cluster 2 patients. It was demonstrated that nitric oxide (NO) induced the apoptosis of neutrophils (Ward et al., 2000). A previous study showed that a large amount of nitrogen species was produced when neutrophils were dead, which is important for their effect in cancer (Pérez-Figueroa et al., 2021). Univariate and multivariate Cox analyses proved that a high density of neutrophils was significantly associated with worse OS.

Existing researches show that neutrophils play a controversial role in the immunotherapy (Deng et al., 2021). In some studies, neutrophils in the immune microenvironment have a negative impact on immunotherapy in NSCLC. Neutrophil Extracellular Traps Promote T Cell Exhaustion in the in NSCLC (Kaltenmeier et al., 2021). In other study, PD-L1+neutrophils reduce T cell cytotoxicity and COX-2 inhibitor can lower PD-L1+neutrophil and improve T cell cytotoxicity (Deng et al., 2021). However, in recent study, diversity and plasticity of tumor-associated neutrophil TAN were revealed and HLA-DR+CD74^+^ neutrophils were positively correlated with the prognosis of several cancer types (Wu et al., 2024). dcTRAIL-R1+ neutrophils are mainly located in the glycolytic and hypoxic niche of the tumor core, highly express vascular endothelial growth factor α and promote angiogenesis and tumor progression (Ng et al., 2024). However, we only observed the prognostic effect of neutrophils in patients with KEAP1 mutation in LUADs. We speculated that this might be related to the effect of KEAP1 mutation on neutrophil typing and differentiation.

The mechanism of neutrophil assembly and the actions of neutrophil effectors in lung tumours are still unknown. It is suggested that neutrophils, rather than other immune cells, may play a crucial role in the tumour immune microenvironment of LUAD patients with KEAP1 mutation. Furthermore, we conducted Immunohistochemical staining and revealed that high density of neutrophile in KEAP1-MT group seemed be related with longer PFS.

We analysed drugs sensitivity between high- and low-KEAP1 expression groups in LUADs from TCGA cohort and found that LUADs with low-KEAP1 expression were more sensitive to ALCAR, AKT inhibitor, AZD6482, AZD7762, Bexarotene, BMS.708.163, BX795 and DMOG (supplementary figure 2). ALCAR was reported increased the levels of antioxidant proteins in human lens epithelial cells (Yang, Yang, and Cao, 2015). However, there is no report about application of ALCAR in lung cancer. Similarly, AKT inhibitor was used in lung cancer with KEAP1 mutation through interaction of cellular antioxidant pathway (Fan et al., 2023) and could assist to kill tumor cell (Dai et al., 2013). We did not find relevant reports that AZD6482, AZD7762, Bexarotene, BMS.708.163, BX795 and DMOG were used in lung cancer with KEAP1 mutation. These drugs may have adjuvant therapeutic effects in patients with KEAP1-mutated lung cancer and need further exploration.

We further analysed the differences in commutations in LUADs with KEAP1 mutations between cluster 2 and clusters 1 and 3 patients. Commutation of KRAS with KEAP1 was significantly more abundant (*p* < 0.01) in cluster 2 patients than in patients in clusters 1 and 3. Kathryn C. Arbor et al. also reached a similar conclusion in a retrospective study of 550 patients (Arbour et al., 2018), indicating that KRAS commutated with the KEAP1 mutant may be a predictor patient prognosis. Conversely, commutation of TTN with KEAP1 was less frequent in cluster 2 patients than in patients in clusters 1 and 3. In a previous study, Cheng X et al. demonstrated that TTN missense mutation was related to longer OS in LUSC patients, while no statistical significance was found in LUAD patients (X. Cheng et al., 2019). However, our results showed that TTN commutated with KEAP1 frequently occurred in favourable prognosis subgroups (clusters 1 and 3) and not in the worse prognosis subgroup (cluster 2) (*p* < 0.05), which may provide new evidence of prognostic factors for LUAD patients with TTN mutations.

There are still several limitations in our study: 1) our data were from TCGA and single-center retrospective study, which may cause bias. 2) Our analysis of OS did not distinguish different treatment modalities in TCGA, which might lead to various results. To verify our results, large-scale research should be conducted in multiple centres based using a greater amount of data. 3) The tumour immune microenvironment in our study was defined by RNA-seq data rather than tumour specimens.

## Conclusion

First, we found that compared to that of patients with LUAD or LUSC with wild-type KEAP1, LUAD or LUSC patients with KEAP1 mutation had a suppressed tumour immune microenvironment, including a decreased density of a suite of CD8^+^ and CD4^+^ T lymphocytes, macrophages, and B lymphocytes, which was observed not only in LUAD but also in LUSC patients. Second, LUAD patients with mutant KEAP1 underwent immunotherapy had worse PFS than with wild-type KEAP1 in CZHR cohorts. Despite the OS of LUAD and LUSC patients was not significantly different between patients with mutant and wild-type KEAP1 in TCGA. Third, the increased density of neutrophils in the tumour immune microenvironment in LUADs with mutant KEAP1 was closely related to worse OS, which means neutrophils may play a more critical role in the prognosis of LUAD patients with mutant KEAP1 than T and B lymphocytes. Accordingly, potential therapy by suppressing neutrophils in patients with KEAP1-mutated LUADs might provide a better prognosis. Fourth, KEAP1 commutated with KRAS may predict a worse prognosis, and TTN commutated with KEAP1 may become a predictor of favourable prognosis. Lastly, the nitrogen metabolism pathway might play a crucial role in the effect of neutrophils in LUAD patients with mutant KEAP1.

## Data Availability

The datasets presented in this study can be found in online repositories. The names of the repository/repositories and accession number(s) can be found in the article/Supplementary material.

## Abbreviations

KEAP1, Kelch-like ECH-associated protein 1; OS, Objective survival; LUAD, Lung adenocarcinoma; LUSC, Lung squamous carcinoma; TCGA, Cancer Genome Atlas; Th2, Type 2 T helper cells; NSCLC, Non-small-cell lung cancer; TME, Tumour immune microenvironment; BOR, Best of response; TPM, Transcript per million; PFS, Progression-free survival.
