# Supplementary material for: Neutrophil in the suppressed immune microenvironment: Critical prognostic factor for lung adenocarcinoma patients with KEAP1 mutation
Source: Front Genet. 2024 Jun 19;15:1382421. doi: 10.3389/fgene.2024.1382421 (PMC11220125; doi:10.3389/fgene.2024.1382421)
Supplement: Supplementary file 1 [file Table1.pdf]

Supplementary table 1. characteristics of patients with squamous carcinoma of lung in CZHR cohorts

Characteristics		KEAP1 wt(n=448)	KEAP1 mt(n=50)	Overall(n=498)
Age (%)		67.36	66.12	67.24
	>=60	360(80.4%)	39(78%)	399(80.1%)
	<60	79(17.6%)	10(20%)	90(18.1%)
	NA	8(1.8%)	1(2%)	9(1.8%)
Sex (%)	Female	105(23.4%)	12(24%)	129(25.9%)
	Male	332 (74.1%)	38(76%)	369(74.1%)
Smoking index		52.13	54.5	52.36
	NA	67	9	76
Stage (%)				
	NA	4 (0.9%)	0	4 (0.8%)
	Stage I	3 (0.7%)	0	3 (0.6%)
	Stage IA	81 (18.1%)	7 (14%)	88 17.7%)
	Stage IB	133 (29.7%)	18 (36%)	151 (30.3%)
	Stage II	3 (0.7%)	0	3(0.6%)
	Stage IIA	59 (13.2%)	5 (10%)	64 (12.9%)
	Stage IIB	85 (19%)	9 (18%)	94 (18.9%)
	Stage III	74 (16.5%)	10 (20%)	84 (16.9%)
	Stage IV	6 (1.3%)	1 (2%)	7 (1.4%)

Supplementary table2. Types of mutants of LUADs with KEAP1 mutation in CZHR cohorts.

Patients	Gene	Types of mutants	PFS (days)	Neutrophil score
Patient 1	KEAP1	P. R442N	360	1%
Patient 2	KEAP1	P. E242K	338	4%
Patient 3	KEAP1	P. A40E	120	5%
Patient 4	KEAP1	P. G417V	300	3%
Patient 5	KEAP1	P. M161I	73	10%
Patient 6	KEAP1	P. A191V	0	12%
Patient 7	KEAP1	P. D422N	0	13%
Patient 8	KEAP1	P. E244K	0	15%
Patient 9	KEAP1	P. G378D	0	25%
Patient 10	KEAP1	P. E307K	0	10%
