# Supplementary material for: Neutrophil in the suppressed immune microenvironment: Critical prognostic factor for lung adenocarcinoma patients with KEAP1 mutation
Source: Front Genet. 2024 Jun 19;15:1382421. doi: 10.3389/fgene.2024.1382421 (PMC11220125; doi:10.3389/fgene.2024.1382421)
Supplement: Supplementary file 2 [file Image1.pdf]

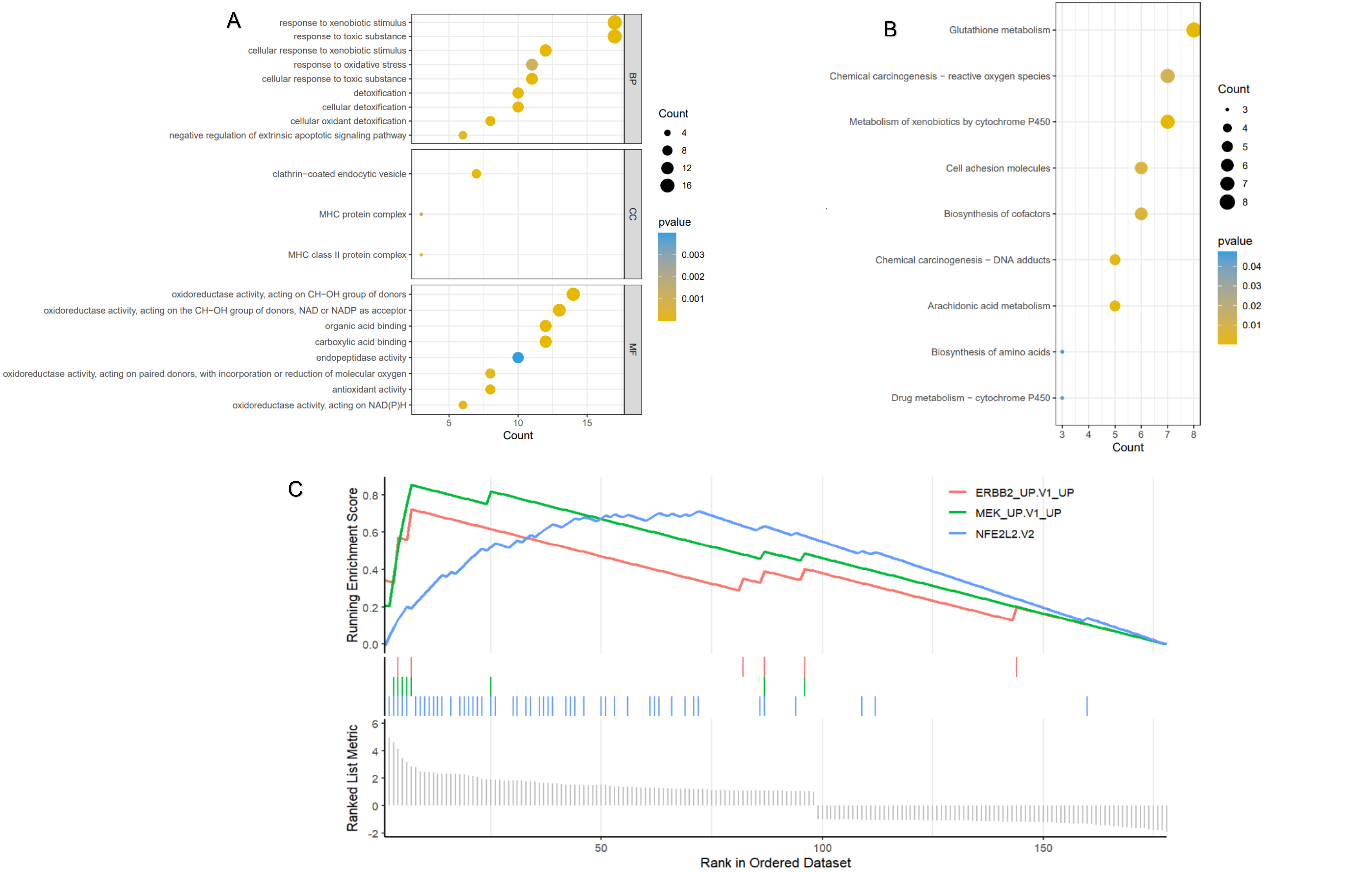

Supplementary Fig.1. The enrichment and analysis of related differential genes between KEAP1 wt and KEAP1 mt. A. Enrichment of MF (molecular function), BP (biological process) and CC (cell component) were conducted according to GO (gene ontology). B. Enrichment of Gene pathways were conducted according to KEGG (Kyoto Encyclopedia of Genes and Genomes). C. GSEA (Gene set enrichment analysis) was conducted between KEAP1 wt and KEAP1 wt.

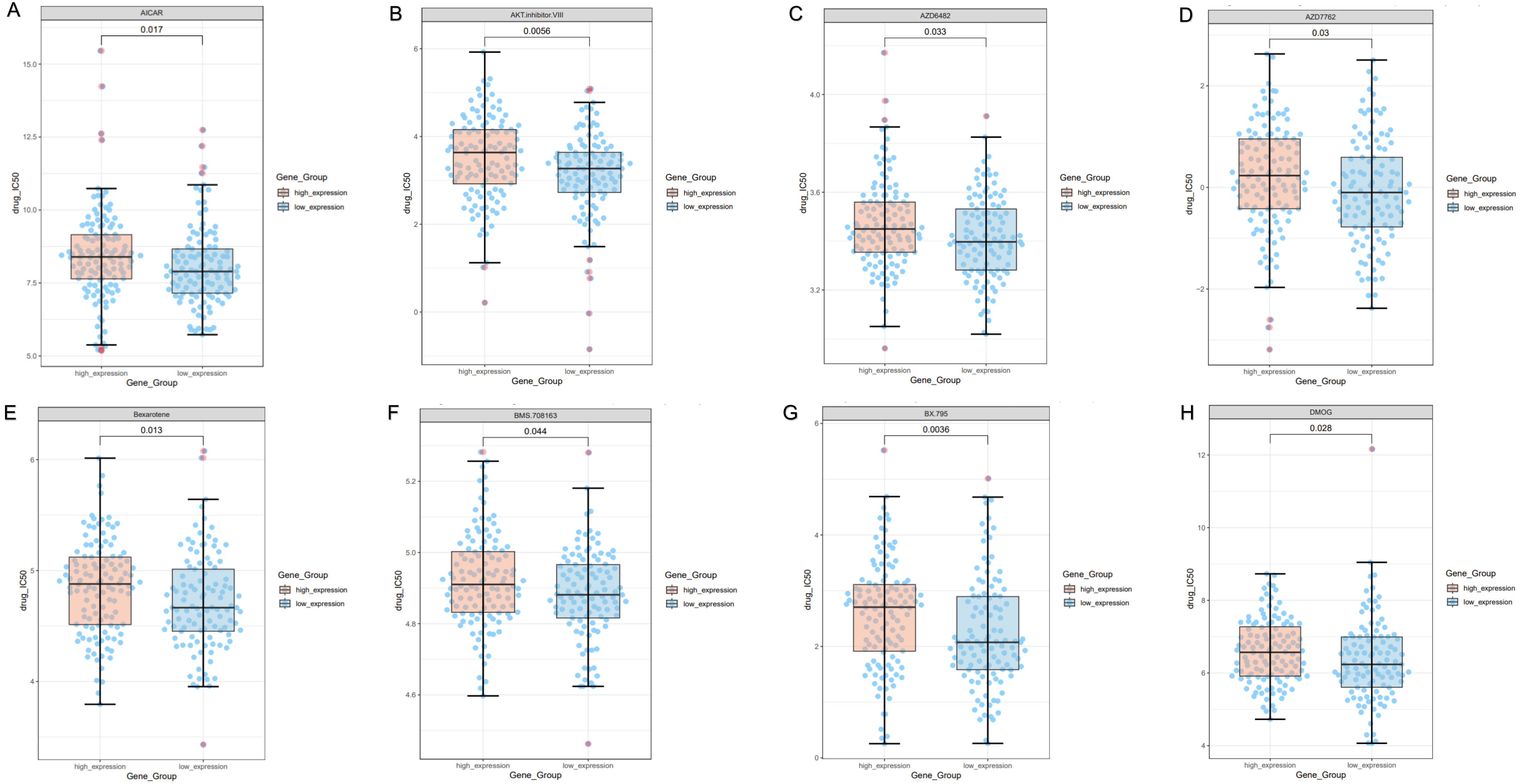

Supplementary Fig.2. Boxplots of the comparison of IC50 of drugs between high- and low-KEAP1 expression groups in LUADs from TCGA cohort. (analysed by https://smuonco.shinyapps.io/CADSP/)
